# The Impact of Engineered Silver Nanomaterials on the Immune System

**DOI:** 10.3390/nano10050967

**Published:** 2020-05-18

**Authors:** Neethu Ninan, Nirmal Goswami, Krasimir Vasilev

**Affiliations:** 1 Unit of Science, Technology, Engineering and Mathematics (STEM), The University of South Australia, Mawson Lakes, SA 5095, Australia; neethu.ninan@unisa.edu.au (N.N.); ngoswami@immt.res.in (N.G.); 2 Future Industries Institute, University of South Australia, Mawson Lakes, SA 5095, Australia; 3 Materials Chemistry Department, CSIR-Institute of Minerals and Materials Technology, Acharya Vihar, Bhubaneswar 751013, India

**Keywords:** silver nanomaterials, immune cells, pro-inflammatory, anti-inflammatory, implants

## Abstract

Over the last decades there has been a tremendous volume of research efforts focused on engineering silver-based (nano)materials. The interest in silver has been mostly driven by the element capacity to kill pathogenic bacteria. In this context, the main area of application has been medical devices that are at significant risk of becoming colonized by bacteria and subsequently infected. However, silver nanomaterials have been incorporated in a number of other commercial products which may or may not benefit from antibacterial protection. The rapid expansion of such products raises important questions about a possible adverse influence on human health. This review focuses on examining currently available literature and summarizing the current state of knowledge of the impact of silver (nano)materials on the immune system. The review also looks at various surface modification strategies used to generate silver-based nanomaterials and the immunomodulatory potential of these materials. It also highlights the immune response triggered by various silver-coated implantable devices and provides guidance and perspective towards engineering silver nanomaterials for modulating immunological consequences.

## 1. Introduction

Engineered nanomaterials have witnessed a dramatic growth in the last few years to meet the growing demand for novel technologies in both the scientific and industrial sectors [1,2,3,4]. The possibility to manipulate the unique features of these materials at the nanoscale through advanced physical, chemical, and engineering approaches brings an additional dimension of interest [5,6,7,8,9,10]. These advances have resulted in a vast library of nanomaterials having many unusual properties [11,12,13,14]. Some of the engineered nanomaterials have been widely used in a broad range of consumer products in fields such as optics [15], electronics [16], personal care [17], and medicine [18]. Examples of such materials include metal nanoparticles (NPs), metal oxides, semiconductors, and/or carbon-based nanomaterials and many others [19,20,21,22,23].

Among those engineered nanomaterials, silver (Ag) nanomaterials are by far some of the most explored because they offer a unique spectrum of physiochemical and biological properties [24,25,26]. With the advancement of nanotechnology, it is now possible to design and produce highly stable silver nanomaterials with tunable size, shape, and surface structure [27,28,29]. For example, plasmonic silver nanoshells were developed as advanced diagnostic tools using various metabolic markers [30]. Metallic nano-architectures of silver and gold with excellent antibacterial properties and biocompatibility was another topic of research interest [31,32,33,34,35]. Spherical silver nanoparticles (AgNPs) from proteinaceous pigment such as phycocyanine have been shown to have anti-cancer activity against breast cancer cell lines [36]. 

Without a doubt, the tremendous interest in silver nanomaterials over the last three decades has been driven by the capacity of silver to kill pathogenic bacteria [37]. This phenomenon is not new, the antibacterial properties of silver have been known since the antiquity and documented in the writings of early civilizations [38]. However, recent advances of nanoscale fabrication techniques allowed for the incorporation of silver in a broad range of consumer products, including medical devices, paints, cosmetics, toys, textiles, water purification technologies, cleaning agents, and many others [39,40,41,42,43,44]. The vast utilization of silver nanomaterials in those applications requires an understanding of how these materials interact with the immune system. On the one hand, knowledge of the immunomodulatory role of silver nanomaterials is vital for the rational design of medical devices which, in addition to protection from infection, provide favorable physiological responses. On the other hand, the wide use of silver nanomaterials may lead to their release in the environment and cause health concerns through dermal contact, ingestion, or inhalation [45,46,47]. This review article provides a bird’s eye view of recent progress in the understanding of the immune responses to engineered silver nanomaterials. 

## 2. Uptake of Silver Nanomaterials by Immune Cells

**Figure 1:**
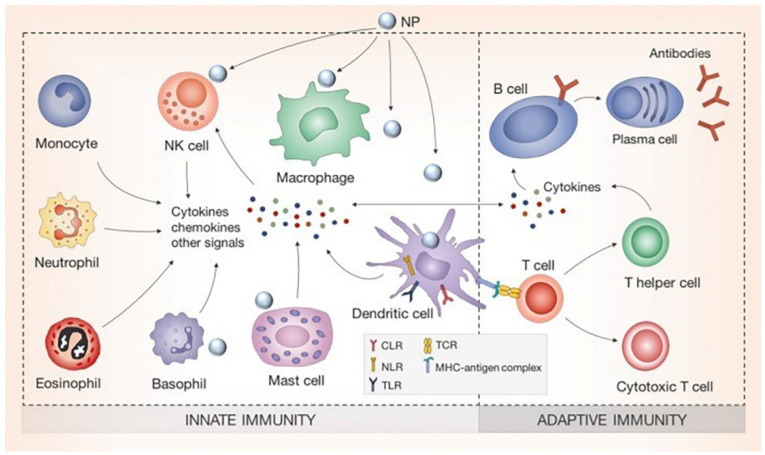
Schematic diagram showing different types of immune cells of the innate and adaptive immunity that may interact with silver nanomaterials. Reproduced from [48], with permission from Elsevier, 2017.

Silver nanomaterials can enter the human body via many pathways, including the respiratory tract, digestive tract, skin, or even through the placenta. Once the NPs overcome the classical barriers of the human body, they reach the bloodstream where they encounter the ‘guard cells’ of the immune system. Immune cells include lymphocytes (B cells, T cells, Natural K cells) and granulocytes (basophils, eosinophils, neutrophils, mast cells, dendritic cells, and macrophages) (Figure 1). Silver can react with these immune cells and incite stimulation or suppression, resulting in various pathological conditions. 

**Figure 2:**
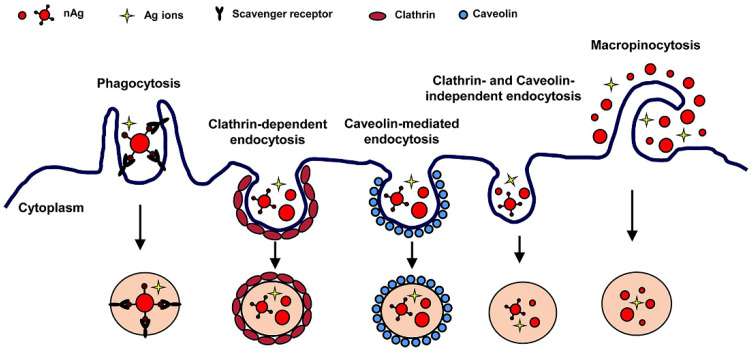
Schematic diagram showing the uptake mechanisms of silver nanomaterials by immune cells. Reproduced from [50], with permission from The Royal Society of Chemistry, 2015.

Mammalian immune cells use five ways to internalize particles: phagocytosis, pinocytosis, clathrin-mediated, caveolin-mediated, and caveolin/clathrin-mediated endocytosis (Figure 2) [49,50]. In phagocytosis, immune cells engulf silver particle to form an internal compartment called phagosome, which then fuses with a lysosome to form a phagolysosome [51]. The enzymes present in phagolysosome will then digest the particles. In pinocytosis, silver is brought out to the mitochondria and then expelled from the cell forming an invagination which is then suspended within a vesicle [52]. Clathrin-mediated endocytosis is a receptor-mediated endocytosis in which cells absorb silver by the inward budding of the plasma membrane containing receptors that specifically bind to silver [53]. Caveolin-mediated endocytosis is a receptor-mediated process in which cells absorb silver by bulb-shaped plasma membrane invaginations driven by integral membrane proteins called caveolin as well as peripheral membrane cavin proteins [54]. 

While the above highlights the possible mechanism of silver uptake, the engulfment of silver nanomaterials is primarily governed by their physicochemical properties. In this context, the following section describes several key parameters that play a crucial role in the resultant immune response by highlighting some recent scientific breakthroughs.

## 3. Role of Physiochemical Properties of Silver Nanomaterials

The physiochemical properties of engineered silver nanomaterials can contribute to both desired and undesired biological response. Understanding of the role that these properties play in modulating immune response can improve safety, which is particularly important for applications in healthcare. Several factors like size, shape, charge, functionalization, etc., can mediate the interaction of silver with immune cells [1]. The size of nanomaterials is a defining feature that determines their in vivo behavior and clearance [55]. In general, particles that are below 5 nm in size are easily cleared by the kidneys while those greater than 100 nm are recognized as foreign materials by immune cells [56,57]. With the decrease in size, the surface area to volume ratio increases enabling particle diffusion into cells [58]. For instance, cellular and nuclear internalization of 5 nm particles is found to be higher compared to 50 nm-sized AgNPs [59]. For example, Park et al. treated human macrophages (U-937) with AgNPs of sizes 4, 20, and 70 nm and found that the smallest particle (4 nm) had the greatest capacity to induce inflammation evident by an increased expression of pro-inflammatory markers [60]. 

The surface charge of nanomaterials also has an impact on cellular uptake. It was demonstrated that positively charged NPs are easily taken up by cells compared to their negative counterparts. This is because the cell membrane is negatively charged facilitating the uptake of oppositely charged particles. For instance, a recent study compared the cellular internalization of polyvinyl pyrrolidone (PVP)-stabilized AgNPs having a net negative charge of −20 mV and polyethyleneimine (PEI)-functionalized AgNPs having a net positive charge of +50 mV [61]. The results indicated that PEI-functionalized AgNPs penetrated the cell membrane easily due to their electropositive nature. Similarly, polyethylene glycol (PEG)-stabilized AgNP/DNA complexes with a zeta potential of 30.5 ± 2.5 mV showed enhanced uptake by immune cell compared to other complexes having a net negative charge [54]. These examples show that the functionalization of silver nanomaterials is an important aspect to be considered to reduce immunotoxicity. It is evident that when particles are functionalized by polyethylene glycol, they can escape the immune system and have a longer circulation time within the body [62]. 

The shape of a NP is another parameter that can influence uptake by immune cells. For instance, nanorods had a longer residence time in the gastrointestinal tract compared to spherical NPs, as nanorods had greater potential to overcome the rapid clearance by the reticuloendothelial system [63]. Nanorods also showed longer circulation time in blood [63]. Moreover, cellular uptake of PVP-stabilized silver nanoprisms and PVP-stabilized spherical AgNPs showed that human mesenchymal stem cells took up more nanoprisms compared to spherical NPs [64]. On the contrary, Hacat cells ingested an equal quantity of both types of silver nanomaterials. This was due to the large interaction area of platelet-like nanosprisms with the cell surface and the bending stiffness of the cell membrane. The more flexible nature of human mesenchymal stem cells membrane appeared to favor uptake of nanoprisms. In contrast, Hacat cells had stiffer cell membrane (high Young’s modulus), and therefore, the energy gain due to the large interaction area of nanoprisms is compensated by the energy spent in deforming the cell membrane to enter inside the cells [64].

It is evident from the above discussion that the physicochemical properties of silver nanomaterials should be carefully considered and assessed in order to be able to accurately modulate their interaction with cellular organelles. These properties determine whether silver nanomaterials can induce inflammation or dampen the immune response. The inflammatory properties of silver nanomaterials are discussed in detail in the following sections.

### 3.1. Pro-Inflammatory Properties of Silver Nanomaterials

**Figure 3:**
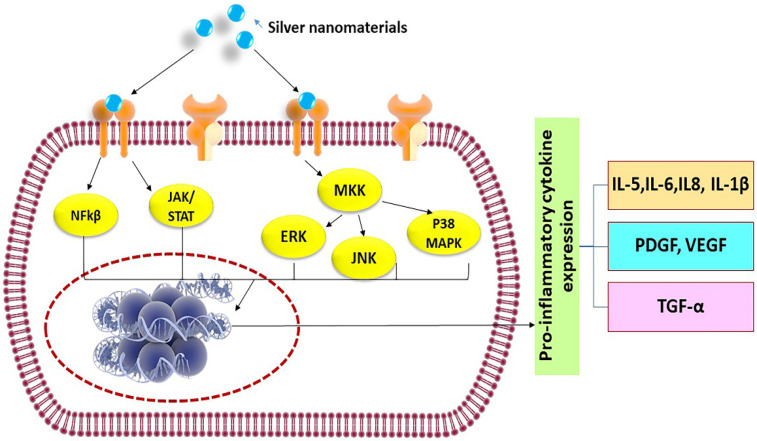
Possible signaling pathways activated by silver nanomaterials to release pro-inflammatory cytokines.

Immune cells recognize silver nanomaterials as foreign particles, thus, any exposure of nanomaterials could trigger a cascade of inflammatory reactions involving the activation of neutrophils, macrophages, and helper T cells, and consequently leading to expression of a large number of cytokines, such as Tumor Necrosis factor-α (TNF-α), Interleukins (IL-1β, IL-6, IL-12, IL-18), etc. [65]. These molecules are part of the normal body natural defense to fight diseases and are utilized in immunotherapies and vaccines. However, the unwanted elevation of a cytokines level in response to nanomaterials may lead to serious side effects, such as systemic inflammation [66]. The possible signaling pathways that may be activated by silver nanomaterials to trigger pro-inflammatory cytokine release are shown in Figure 3 and include Nuclear Factor-kβ (NF-kβ), c-Jun N-terminal kinase (JKK), or Mitogen-activated protein kinase kinase (MKK) pathways. In a normal physiological situation, in order to avert diseases, it is necessary to tightly regulate the production of cytokines and prevent overstimulation of the immune system. Therefore, it is important to conduct a systematic evaluation of the propensity of silver nanomaterials in inducing cytokine release from immune cells as a major parameter for confirming their safety profile. Various in vitro and in vivo assays [67] to explore the immune responses caused by engineered silver materials are discussed in Table 1 and Table 2.

Size-dependent pro-inflammatory consequences triggered by silver nanomaterials can be noticed in several instances. When NPs enter the biological system, there is a greater neutrophil influx in case of particles of sizes below 100 nm compared to their larger counterparts. For instance, it has been demonstrated that smaller sized AgNPs (3–5 nm) could cross the cell membrane of neural cells and induce IL-1β secretion [85]. These AgNPs were internalized by endocytic uptake processes into small cellular vesicles that could release silver and induce generation of ROS and inflammation. They activated immune reaction genes, including CXCL13 and MARCO, which caused release of TNF-α in zebrafish liver cells, thus triggering pro-inflammatory events [85]. In another study, AgNPs of sizes of about 56 nm upregulated the expression of pro-inflammatory cytokines such as IL-1β and IL-6 in human lung epithelial cells (A549) [86]. Giovanni et al. reported the activation of pro-inflammatory responses in macrophages that were exposed to an ultra-low concentration of silver [87]. These studies indicate that particle size is therefore an important factor that drives inflammatory events [88].

Surface modification of nanomaterials can also alter the response of the immune system. It is interesting to note that some of the functionalized AgNPs were found to elicit pro-inflammatory properties. Exposure to PVP-coated AgNPs induced pro-inflammatory cytokine gene expression of TNF-α, IL-1, and IL-6 in both primary blood monocytes and THP-1 cells [89]. They also resulted in the release of IL-1β by the formation of inflammasome [90,91], a multi-protein oligomer produced by myeloid cells during innate immunity. These studies showed that PVP-coated AgNPs could induce an innate immune response which could lead to the risk of inflammatory disease development. In another study, the non-methylated cytosine–phosphate–guanine (CpG)-functionalized silver nanoclusters were found to enhance immune response and were used for cell imaging [70]. CpG dinucleotide is present in bacterial and viral DNA and exhibits immune-stimulatory activities to attacking pathogens. The immune system in mammals recognizes CpG oligodeoxynucleotides via Toll-like receptor-9 and release several pro-inflammatory cytokines, including IL-6 and TNF-α, which can stimulate both the innate and adaptive immunity. CpG-functionalized silver nanoclusters exhibited minimum toxicity and were easily internalized by cells and effectively protected from nuclease degradation. These silver nanoclusters showed an immune-stimulatory effect when combined with CpG, increasing TNF-α and IL-6 production [92]. All these different scenarios explain that the choice of surface modification can determine the fate of a silver nanomaterial entering a biological system.

### 3.2. Anti-Inflammatory and Immunosuppressive Properties of Silver Nanomaterials

Anti-inflammatory cytokines are regulatory molecules that dampen the immune response. Cytokines which can have this role include IL-4, IL-10, IL-11, IL-13, and TGF-β [93]. Silver nanomaterials can be engineered to directly target immune cells and suppress their activity or avoid immune recognition. Regulation of toll-like receptor (TLR) signaling using tactfully designed silver nanomaterials is one approach to combat overpowering inflammatory response. TLRs are non-catalytic receptor proteins found mostly in macrophages and dendritic cells that help in recognizing microbes or foreign particles [94]. AgNPs capped with a monolayer of tiopronin reduced the secretion of IL-6 mediated by TLR ligands without inducing any toxicity [95]. Bergenin-loaded AgNPs and stabilized by gum xantham exhibited inhibitory effects on TLR-2 and TLR-4 and suppressed synovial inflammation in arthritic rats [96]. 

Anti-cytokine approaches using silver nanomaterials are yet another interesting method to reduce inflammation in which interaction between cytokines and their receptors is prevented and cytokine gene expression is reduced. Bioengineered mannan sulphate-capped AgNPs were found to downregulate both TNF-α and IL-6 expression in rats [97]. These were spherical particles with a size of 20 nm and a zeta potential of −32.4 mV. The particles were internalized by murine macrophage cell lines using receptor-mediated endocytosis via mannose receptors. These particles not only reduced inflammation but also accelerated wound healing in rats. A comparative study of nanocrystalline silver and silver nitrate in a porcine model of contact dermatitis showed that nanocrystalline silver-treated pigs had a reduced level of edema and erythema after 72 h and also a decreased expression of pro-inflammatory cytokines compared to animal ammonals treated with AgNO_3_ [82]. This result suggested that nanocrystalline silver could benefit wound healing. In another study, the cytokine level expression in wounds treated with genipin-crosslinked chitosan hydrogels containing silver sulfadiazine nanocrystals showed reduced IL-6 levels [98].

Appropriately designed silver nanomaterials were shown to inhibit inflammatory cell recruitment to the affected tissues, thereby preventing inflammation. This is particularly useful for implant transplantation and drug delivery. AgNPs coated onto the surface of absorbable braided suture using a layer by layer deposition showed remarkable anti-inflammatory property in mice. Immunohistochemistry results and quantitative evaluation showed reduced macrophage infiltration and decreased the production of IL-10, IL-6, and TNF-α [99].

Inhibition of T lymphocytes by silver nanomaterials is another strategy to suppress the immune system. In this context, Côté-Maurais et al. studied the effect of AgNPs on interleukin-2 (IL-2) dependent proliferation of T cells [100]. Upon activation, CD4+ T cells produce IL-2, which is a key mediator of proliferation, differentiation, and growth of effector CD4+ T cells. The authors demonstrated that AgNPs alter IL-2 release by reducing T cell proliferation due to the rise of low-affinity receptors for this cytokine [100]. 

**Figure 4:**
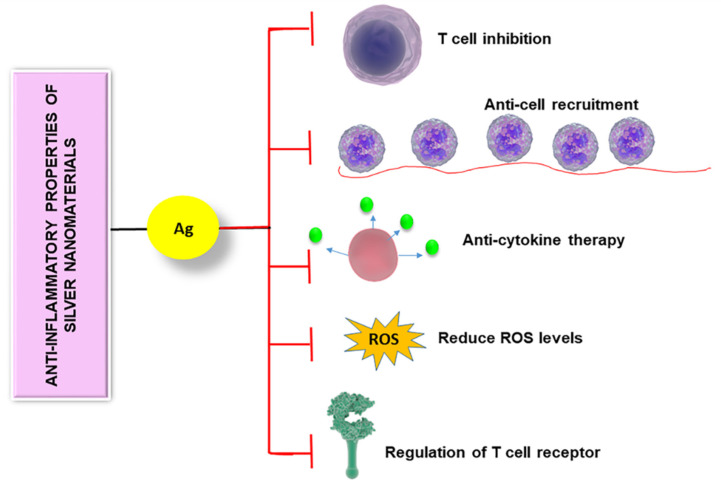
Anti-inflammatory properties of silver nanomaterials are controlled by different mechanisms including T cell inhibition, anti-cell recruitment, anti-cytokine therapy, regulation of T cell receptor, and reducing the level of reactive oxygen species.

Reactive oxygen species act as immune mediators affecting various immune cells [101]. When the level of ROS is elevated, immune cells become dysfunctional which often leads to immunosuppression [102]. Silver nanomaterials can be used to reduce the ROS level, thereby controlling the immune system. Manikandan et al. synthesized AgNPs using an ethanolic extract of rose petals and tested their anti-inflammatory activity on rat peritoneal macrophages in vitro [75]. They observed that exposure of the prepared NPs reduced H_2_O_2_-mediated cytotoxicity in macrophages. They also found a noticeable reduction in the liberation of potent inflammatory mediators such as superoxide anion and nitric oxide upon exposure to NPs. Another group focused on studying the impact of AgNPs on microglial inflammation as microglia are greatly affected in neurodegenerative disorders (Figure 4) [103]. Microglia are macrophage cells that act as the key active immune defense in the central nervous system. They found that after internalization of AgNPs by microglia, non-reactive silver sulphide was formed on the surface of AgNPs. Furthermore, these NPs increased the expression of hydrogen sulphide synthesizing an enzyme called cystathionine-γ-lyase and showed remarkable anti-inflammatory effects, plummeting LPS-stimulated nitric oxide, ROS, and TNF-α levels. These studies reveal the anti-inflammatory activity of various forms of silver nanomaterials. 

### 3.3. Adjuvant Properties of Silver Nanomaterials

Adjuvants are moieties added to a vaccine to increase immune responses towards antigens. They can act in different ways to present antigens to the immune system by either acting as antigen depot or as irritants. Antigen depots present antigen over a greater period of time, thus maximizing immune response [104]. Others act as irritants which increase the body’s immune response [105]. The action of adjuvants is mainly controlled by T and B cells. For instance, Xu et al. evaluated the adjuvant effect of AgNPs both in vitro and in vivo [106]. These negatively charged NPs (−30.6 mV) with a size of ~141 nm were found to increase the serum antigen-specific IgG and IgE levels in mice, indicating that AgNPs elicited CD 4+-mediated immune response. The mechanism of an adjuvant effect is mainly attributed to the activation and recruitment of local leukocytes and macrophages by AgNPs. Asgary et al. prepared spherical AgNPs from leaf extract of Eucalyptus procera having an average size of ~60 nm and a zeta potential of −14 mV [107]. Different amounts of the prepared NPs were added to inactivated rabies virus and injected into the peritoneum of mice. The results showed that the adjuvant effect of AgNPs was enhanced by increasing their concentration and finally reached a plateau at 15–20 mg/kg. The authors proposed a mechanism for the adjuvant effect which includes accumulation and trapping of antigen by AgNPs, which leads to a better regulation of the innate immune system [107]. 

Silver nanomaterials can be used to develop HIV vaccines that can adequately activate critical anti-HIV immunity in a safe manner. Even though some prevailing adjuvants can assist developing immunity by HIV vaccines, they have aftereffects, as they go into host cells along with vaccine delivery; in other words, they are intracellular adjuvants that can damage host cells, ultimately causing cytotoxicity. Liu et al. demonstrated that polyvinylpyrrolidone–polyethylene glycol-modified silver nanorods can be a harmless nanocarrier adjuvant for a HIV vaccine which stays outside the cells but can still trigger immune response [108]. Compared to nanospheres, nanorods cannot be easily taken up by cells. Silver was chosen as the non-carrier adjuvant for HIV vaccine as it can inhibit HIV and can boost antibody and T cell response against ovalbumin. These nanorods improved HIV vaccine-specific IgG responses by raising the titer of IgG3. IgG3 inhibits HIV by binding to Fc receptor and mediated antibody-dependent cellular toxicity. Silver nanorods induced T cells to secrete more IFN-γ, CD107, IL-2, and IL-4. Thus, these nanorods increased critical immunities of HIV vaccine and were not cytotoxic, which is the main problem faced by other adjuvants. However, more research needs to be conducted in the future to exploit the adjuvant properties of silver nanomaterials.

## 4. Immune Response of Silver-Coated Implants

Existing state-of-the-art implant technology makes use of the immunomodulatory properties of silver nanomaterials for various applications. Medical implants are devices located inside the body to replace body parts, deliver medication, provide support, or monitor the functions of the body [109]. Despite of the wide success in the treatment of disease and injury, medical implants suffer from a range of complications and may fail to function [110] due to several obscuring factors such as infections, inflammatory reactions, poor integration into host tissue, etc. [111,112]. An important aspect area of recent development is engineering the surface properties of implantable devices to modulate immune responses and improve function [113,114].

More than half of all implant-related infections are instigated by bacteria colonizing and forming a biofilm on medical device surfaces. Affected medical devices include contact lenses, prosthetic heart valve, hip, knee, and other orthopedic implants, fixation and spinal devices, urinary catheters, intrauterine devices, central venous catheters, and dental implants, just to name a few [115]. The formation of biofilm on medical devices is a serious challenge that leads to huge medical and economic losses. Various strategies have been devised to protect the device from colonization by bacteria and biofilm formation including placing an antibacterial coating on the medical device surface or impregnating with antibacterial agents [115,116]. Antibacterial surface technologies employing silver is one such strategy that has gained enormous attention. In fact, silver in the form of salts, metal films, and NPs have been the dominant component of antibacterial technology over the last two decades [30,37]. Numerous research reports have explored the synthesis of AgNPs by various means [117,118,119], the preparation of hybrid AgNP containing materials [118,120,121,122], as well as the applications of Ag nanomaterials in developing antibacterial coatings [123,124,125,126,127,128]. 

In the context of the widespread use of silver-facilitated antibacterial technologies, it is important to understand the immune response to such devices. This is because only very slight changes in biomaterial chemistry and structure can lead to significant inflammatory consequences. Material chemistry of silver-coated implants can affect the immune response and play a significant role in regulating the release of cytokines from monocytes and macrophages [129]. The cytokine release upon adherence to implant surfaces determines whether immune cells view the implants as a pro-inflammatory or an anti-inflammatory stimulus. Silver-coated medical devices when introduced into the body can elicit an immune response. Therefore, for any translational application of implantable biomaterials, an important step is to understand their impact on the immune system. In the following section, we discuss key silver-coated implantable devices and the resultant immune responses.

### 4.1. Immune Response of Silver-Coated Dental Implants

Dental implants are commonly used to replace decayed or missing teeth [130]. Like natural teeth, they can become infected and fail to perform if left untreated. Peri-implantitis is a serious complication in dental implants as it can result in degeneration of jawbones [131]. When dental implants are introduced into our body, there is a race between the body’s own cellular defense and infectious pathogens. If bacteria win the race, a biofilm can form on the surface of the implant and increase resistance to antibiotics. This may result in a destructive inflammatory reaction or peri-implantitis, which can result in bone atrophy. Periodontitis is another condition that leads to soft tissue inflammation resulting in alveolar bone loss around natural teeth instigated by bacteria [132]. It may result in tooth loss if left untreated. Lost teeth are replaced by costly dental implants, crowns, and screws. Several strategies are developed to render the surface of dental implants antibacterial to prevent periodontitis and peri-implantitis [133]. Nano and micron scale silver-based coatings can prevent infection and associated inflammation and improve the long-term effectiveness of dental implants. For instance, a new type of implant coating (DentaPlas) composed of two plasma polymer layers surrounding a central layer of AgNPs was developed recently [134]. The product is claimed to not only release silver to kill bacteria but also to promote cellular growth by overcoming the immune response of the body. In another study, silver–platinum alloys were coated onto stainless steel devices which were then used as braces and archwires in dentistry [135]. Platinum improves hardness and wear resistance while silver provides the antibacterial and anti-inflammatory activity [135]. Electroplating is yet another technique used to coat silver onto orthodontic brackets to provide protection against bacteria and reduce inflammation [136]. Nanosilver was coated on standard orthodontic brackets by physical vapor deposition technique at a thickness of 1 µm. The in vivo studies in Wistar rats showed that the coated devices exhibited antibacterial and antifungal properties with no adverse inflammatory response [137]. Another study revealed that titanium implants coated with nanocrystaline silver, copper, and bismuth using pulsed magnetron sputtering reduced biofilm formation and peri-implant inflammation [138]. Nanosilver coatings have been also applied on polymeric devices for dental applications. Polyetherether ketone (PEEK) is a thermoplastic polymer with good mechanical properties such as rigidity, high strength, corrosion resistance, and biocompatibility and is used for the manufacture of healing cap and implant abutments [133]. PEEK coated with nanosilver using magnetron sputtering exhibited good antibacterial properties against *Streptococcus mutans* (*S. mutans*) and *Staphylococcus aureus* (*S. aureus*) and showed no cytotoxicity [133]. Schwass et al. have developed silver alginate gel formulation that consists of AgNPs with a size of 7 nm. It was applied to gum pockets in sheep with gum disease and was found to reduce inflammation and promote healing around teeth and implants [139].

Despite the demonstrated benefits of applying silver-based coating of dental implants, one cannot ignore the potential immunotoxicity of the metal ions and NPs if used in higher dosages. For instance, silver solders which are used to connect support wires in orthodontic appliances have been found to release a high amount of silver ions that result in immunotoxicity [140]. In another study, AgNPs with an average size of 5 nm were found to induce an inflammatory response and were cytotoxic at a concentration of 25 µg/mL [141]. These studies demonstrate that silver coatings and nanomaterials must be used within a therapeutic window that allows to minimize biofilm growth but does not lead to adverse inflammatory consequences and/or cytotoxicity. 

### 4.2. Immune Response of Silver-Coated Bone Implants

**Figure 5:**
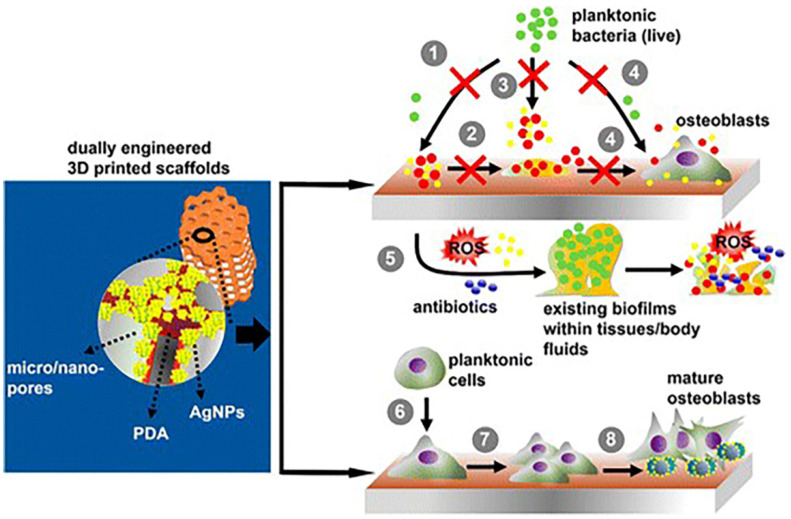
Schematic showing 3D printed titanium implant incorporating spherical silver nanoparticles (AgNPs) capable of eradicating biofilm (1–4) and promoting bone repair (5–8). Reproduced from [149], with permission from American Chemical Society, 2016.

Osteomyelitis is an infection caused primarily due to bacteria or fungus entering the bone tissue from the bloodstream via open wounds or surgery [142]. This condition is found in people of all ages and is very destructive because the disease-causing microbes can damage healthy tissues and progress rapidly. Despite advances in operative techniques and the strict applications of antibiotics, osteomyelitis remains a great challenge and is expensive to treat. Osteomyelitis is very often seen in patients who are subjected to implant surgery. It is challenging to treat implant-associated infection, which in severe cases can lead to amputation and patient death. Current treatment strategies involve local and systemic antimicrobial therapies and surgical debridement [143]. Silver ions were employed to treat chronic osteomyelitis with improved efficiency [144]. Lu et al. developed titanium nanoscaffolds containing silver-doped nano-hydroxyapatite/polyamide-66 materials for the treatment of osteomyelitis that exerted potent anti-inflammation and anti-bacterial effects in vivo and promoted bone formation at the lesion sites of osteomyelitis. At 12 weeks after debridement surgery, inflammatory cells were rarely observed in the scaffold materials; rather, new trabecular bone formation and neovascularization were observed at the bone scaffold interface [144]. Intelligent hybrid implant materials composed of AgNPs embedded within diamond-like carbon surfaces were able to prevent bacterial colonization and facilitate the growth of mammalian cells, including endothelial cells and osteoblasts. The study noted that the human monocytic cell line, THP-1, showed the lowest tolerance to silver nitrate at concentrations of 10 µM [120]. Funao et al. coated hydroxyapatite film with ionic silver via inositol hexaphosphate chelation and evaluated their effectiveness in a bioluminescent murine osteomyelitis model [145]. They showed a reduction in the levels of IL-6 and CRP protein in blood serum and reduced infection. Histology analysis did not show any infiltration of neutrophils in the region of interest, suggesting lack of inflammation. In another study, growth factors and silver were incorporated into hydroxyapatite coatings of metallic implants and were further implanted into the femur of rabbits. The results revealed that the coatings favored bone formation in vivo with reduced inflammation [146]. A different approach using a combination of silver, hydroxyapatite, and titanium nano-coatings on the surface of titanium alloy implants demonstrated a reduction of bacterial biofilm by 97.5% and supported bone healing with decreased inflammation [133]. Bioinspired anchoring of AgNPs onto titanium implants gave rise to anti-corrosive and anti-bacterial coatings on bone implants. These hierarchical Ag/Ti implants promoted the proliferation and differentiation of osteoblasts [147]. In another study, biotransformation of silver released from AgNP-coated titanium implants was found to regenerate bone in adult male Wistar rats [148]. 3D printed porous titanium surface engineered with micro/nano topology incorporating AgNPs were found to reduce infection and promote bone growth and were suggested as another promising approach to control immune response provoked rejection (Figure 5) [149].

Periprosthetic infection is another serious complication seen after the implantation of tumor endoprosthesis [150]. Such megaprostheses are used to reconstruct large bone defect. Hardest et al. replaced diseased bone with titanium or silver-coated megaprostheses in 98 patients with sarcoma or giant cell tumor in the proximal tibia. They found that silver-coated megaprosthess were able to reduce infection and inflammation much more efficiently compared to standard titanium devices [151]. Thus, there is compelling evidence that when used appropriately, silver coatings and NPs can efficiently control inflammation and infection with orthopedic implants.

### 4.3. Immune Response of Silver-Coated Wound Dressings

Wound healing is a complex phenomenon comprised of a series of successive events to repair injured tissue [152,153]. The immune system has a vital role in regulating the wound healing process and fighting against foreign bodies and pathogens [154]. Bacterial load on the surface of the wound can activate pro-inflammatory signaling pathway. Biofilm is a major component of unmanageable chronic wounds and is identified as one of the reasons for the recurrent failure of antiseptics and antibiotics in these circumstances. Ionic silver is an effective and broad-spectrum antiseptic used to reduce wound infections, but like antibiotics, its effectiveness is limited in eradicating biofilms. Researchers found that silver containing hydrofiber dressings considerably reduced polymicrobial biofilm and subdued wound inflammation in a rabbit ear wound biofilm model [155]. The silver incorporated dressing was able to transform established biofilm to planktonic bacterial population which are more susceptible to silver. The clinical efficacy was confirmed on 42 patients with recalcitrant venous leg ulcers and 113 recalcitrant wounds with varied etiology [155]. Silver sulfadiazine-loaded chitosan composite sponges were used to treat acute burn wounds in Kunming male mice [98]. The histopathology results showed a reduction in the number of macrophages in burns treated with the silver-loaded sponge which contributed to improved healing [98]. DeBoer et al. designed a silver (I) complex incorporated within carboxymethyl cellulose hydrogel that allowed slow release of silver. The hydrogel increased the bioavailability of Ag^+^ and achieving MIC values of 4.7 and 2.3 µg/mL against *S. aureus* and *Escherichia coli*, respectively [156]. Evaluation of Acticoat silver-based dressings was conducted in a rabbit model with muscle injury contaminated by *S. aureus* [157]. The molecular biology results showed increased regeneration and mitigated inflammation. The gene analysis indicated a switch from inflammation to wound healing by the downregulation of MCP-1 which contributed to better wound healing [157].

**Figure 6:**
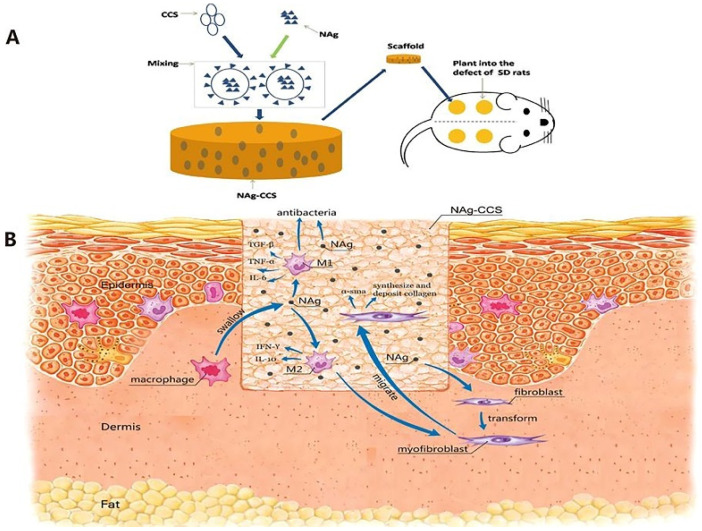
Schematic illustration showing (**A**) the implantation of AgNPs-loaded collagen/chitosan scaffold and (**B**) the possible mechanism of accelerating cutaneous wound healing. Reproduced from [158] with permission from Springer Nature, 2017.

AgNPs-loaded collagen/chitosan scaffolds promoted wound healing in vitro and in vivo by regulating fibroblast migration and macrophage activation (Figure 6) [158]. The in vivo studies on Sprague Dawley rats showed no evidence of inflammatory reactions and reduction of the expression of CD-68, IL-6, and TGF-β. The scaffolds upregulated the expression of anti-inflammatory cytokines, including IFN-γ and IL-10 [158]. CD-68 is a marker expressed by macrophages and is a direct sign of infiltration of inflammatory cells. The overexpression of CD-68, IL-6, and TGF-β in wounds leads to delayed wound healing. IFN-γ plays a vital role in tissue remodeling of wounds by reducing collagen production and acts as an inducer for activation of the M2 pro-healing macrophage phenotype. An Alginate/AgNPs composite sponge was also found to lower pro-inflammatory cytokine levels [159]. The oral administration of AgNPs was shown to enhance inflammatory response in mice by increasing the expression of IL-1 and IL-6. However, reverse effects were observed when AgNPs were embedded in alginate matrices. Abdel-Mohsen et al. fabricated AgNPs/hyaluronan bionanocomposite and used the in-wound dressing for chronic ulcers. The in vivo studies in diabetic rat models showed accelerated wound healing and reduced inflammation [160]. Nanocrystalline silver-coated wound dressings were found to induce anti-inflammatory activity in a contact dermatitis model [82]. The extensive in vivo and in vitro studies of silver-loaded wound dressing demonstrate that when presented in the appropriate manner, silver can have both anti-inflammatory and anti-bacterial properties and promote wound healing.

### 4.4. Immune Response of Silver-Coated Vascular Catheters

**Figure 7:**
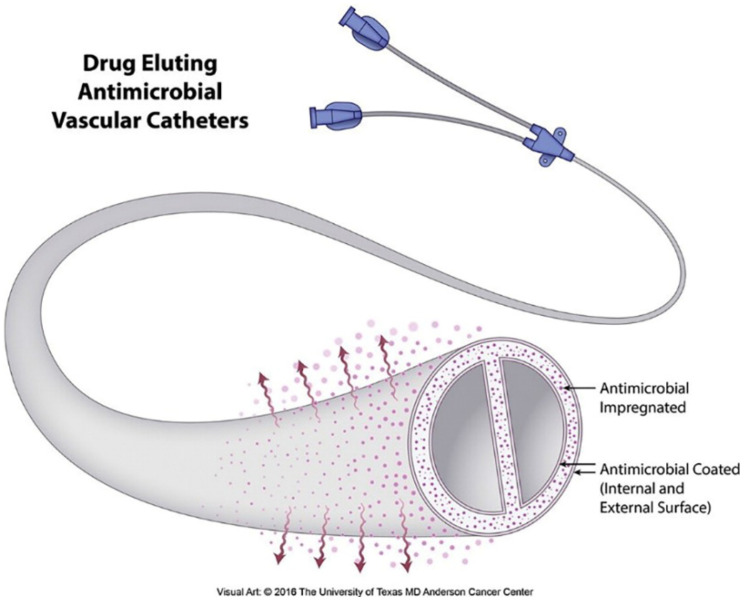
Schematic diagram showing the concept of a drug-releasing antimicrobial vascular catheter. Reproduced from [163] with permission from Elsevier, 2017.

Vascular catheters are essential tools in current healthcare for providing medical care to patients [161]. However, these devices pose a considerable danger of severe bloodstream infections, often leading to major complications and even patient death. The situation with dialysis catheters is especially troublesome where bacteremia is termed “a problem of epidemic proportions” [162]. Both the peripheral and interior surfaces of catheters are prone to infections. Drug releasing vascular catheters play a vital role in reducing catheter-related bloodstream infections (Figure 7) [163]. Over two decades, hospitals have used vascular catheters with eluting combinations of antiseptics and antibiotics. Examples are catheters loaded with antiseptics like silver and chlorhexidine, and antibiotics such as sulfadiazine [164].

Another innovation were silver–carbon–platinum (SCP) catheters which release Ag^+^ ions in physiological media by an electrochemical reaction. The higher electrochemical reduction potential of platinum attracts electrons from silver forming Ag^+^ ions. The role of carbon is to prevent ionic charge build-up between silver and platinum. A study has been conducted to compare the infection rate of SCP catheters and rifampicin–minocycline (RM)-coated catheters [165]. Both devices had low rates of catheter-related bloodstream infections, but the SCP catheters were clinically more effective as they also reduced inflammation.

Silver bound to zeolites (a biocompatible aluminosilicate) is being also used in catheters. Central vascular catheters impregnated with silver zeolite formulation were found to reduce catheter-related infection in adults under critical care [166]. Silver zeolite-loaded umbilical catheters were also found to reduce catheter-related bloodstream infections and subdued inflammation in infants [167]. Silver-heparin coatings of catheters were found to reduce thrombogenicity as well as prevent infections. Schneider et al. tested the efficiency of grafts coated with collagen/silver along with gelatin-sealed grafts both soaked in rifampin to prevent bacterial infection in a dog model. The results showed no sign of inflammation and that the grafts were effective against *E. coli* and *Methylene resistant S. aureus* (MRSA) [168]. Dispersing AgNPs in catheters is another approach to continually utilize silver ions to protect from infections [169,170]. These devices were demonstrated not only to protect from infections but also to have good biocompatibility. Despite the initial concerns about using silver with vascular catheters, research and practice have demonstrated significant potential in protecting patients from devastating infections. 

## 5. Bio-Modifications of AgNPs

When nanomaterials are exposed to the biological fluids, extracellular proteins adsorb on their surface forming the so-called protein corona that can critically impact cellular interaction. Protein corona is composed of an inner layer of strongly bound proteins (‘hard’ corona) and an outer layer composed of loosely bound proteins (‘soft corona’). Based on the nature, amount, and orientation of bound proteins, the immune response can be activated or suppressed. It was demonstrated that surface curvature of AgNPs can influence protein corona formation. Citrate and PVP-stabilized AgNPs exhibited amplified binding of proteins with an increase in their size, suggesting the impact of surface curvature on protein corona [171]. A recent study also revealed that protein corona has a strong modulatory effect on the biotransformation of AgNPs. The researchers found that silver ions released from AgNPs were trapped in the protein corona and were converted to silver sulphide nanocrystals, thereby minimizing the toxicity of AgNPs [172]. In another study, it was evaluated that AgNPs capped with alkanethiol were more resistant to protein adsorption compared to citrate-capped AgNPs, and hence exhibited reduced cellular uptake and toxicity [173]. 

Bio-modifications of silver nanomaterials is a strategy that can control protein corona formation and could be potentially efficient in modulating interactions with the immune system. This strategy involves attaching to the surface of the NPs appropriate biological molecules, such as proteins, lipids, or other ligands, such as sugars or starches of polyethylene glycol (PEG). For example, PEG is well regarded for its capacity to regulate protein adsorption, and in this way, the interactions of a material surface and the innate immune system [174]. PEG-modified nanomaterials demonstrated improved plasma half-life, decreased immunogenicity, and reduced clearance by the reticuloendothelial system. PEG-stabilized AgNPs were used as an efficient non-viral carrier in gene therapy with minimal toxicity [54]. AgNPs-impregnated PEG/chitosan hydrogels enhanced wound healing and reduced inflammation in diabetic rabbits [175]. PEG/graphene oxide/AgNPs composites exhibited no cell toxicity and excellent antibacterial properties [176].

Stable protein conjugates of AgNPs can be prepared by passive adsorption and covalent binding. In the case of passive adsorption, electrostatic and hydrophobic interactions are utilized between the surface of colloidal silver and protein molecule at a pH close to the isoelectric point of the protein to be conjugated [177]. Stability of NPs also depends on protein loading. If the amount of proteins adsorbed on the surface is not sufficient, aggregation can occur upon the addition of electrolytes. In some cases, passive adsorption can lead to a disturbance in the protein tertiary structure which may affect their functionality. This can be avoided by covalent coupling of protein onto modified silver nanomaterials with carboxyl groups by means of a PEG linker [178]. Such conjugations are possible based on carbodiimide chemistry. AgNPs protein conjugates were reported with bovine serum albumin [179], β-glucosidase extracted from bacterium [180], high-density lipoprotein [181], human serum albumin [181], casein [182], etc. An exciting future opportunity is to conjugate silver nanomaterials with recombinant proteins as such proteins can strongly modulate the immune response [183]. Another strategy for bioconjugation of biomolecules is this developed by Vasilev and co-workers based on plasma polymer coatings deposited from oxazoline-based precursors [184,185]. This strategy has numerous advantages over other methods. Plasma polymer coating with good adhesion to the surface can be deposited to almost any type of substrate material without the need on surface prenotification [186] as in the case of Layed-by-Layer [187], for example. Oxazoline coatings deposited from plasma retain a population of intact oxazoline rings which can bind carboxyl acid groups (available in biomolecules such as proteins and antibodies) in a fast, one step, click type reaction and have been already used in diagnostic technologies [188,189]. Even more interesting, this type of coatings were found to inhibit biofilm growth and reduce expression of pro-inflammatory cytokines from macrophages [190].

Glycosylation is a post-translational modification of eukaryotic proteins that affect their functions, including immunogenicity, folding, and stability [191]. Immune modulation and cell-specific targeting are made possible because the lectin receptors in cells can recognize glycan protein structures. For example, myeloid C-type lectin receptors (CLR) are targets capable of triggering an immune response [192]. Glycan-based CLR targeting has gained more attention compared to antibody mediating targeting because of lower immunogenicity and the ability to target many CLR concurrently. However, targeting efficacy is affected by several factors, such as surface charge, size, conjugation method, and ligand density. For CLR targeting, NPs can be decorated with multivalent glycans and serve as promising carriers. The outer carbohydrate shells of these NPs enable them to escape the surveillance of macrophages and allow specific binding to lectin receptors on tumors. The NPs were stabilized using potato starch and chitosan without affecting their bactericidal properties [193]. Kennedy et al. have prepared thiol-functionalized carbohydrates, including glucose, galactose, and mannose, and treated them with citrate-capped silver NPs [194]. Galactose and mannose-functionalized silver NPs were not toxic against neuronal-like cell line (Neuro-2A) and hepatocyte cell line (HepG2). The toxicity in case of glucose was correlated to intracellular oxidative stress, measured as protein carbonylation [194]. Several studies reported NPs decorated with N-linked high mannose glycan clusters, galactofuranose, mannosylated starch nanocarriers, mannose, glucose, and fucose [195]. These results open new possibilities to modify the bioactivity of silver nanomaterials by using various types of carbohydrates.

Lipids are hydrophobic or amphiphilic entities, present in oils, fatty acids, and waxes and are found in various forms [196]. Lipids were used for the encapsulation of silver nanomaterials as they were expected to improve biocompatibility and reduce undesirable inflammatory responses since the outer structure mimics the membrane of biological cells [197]. The physicochemical properties of lipid-coated particles, such as size, lipid composition, surface characteristics, charge, geometry, dosage, and mode of administration, can all affect the way they modulate immune response [198]. For example, lipid nanoparticles (LNPs) with large sizes between 90 and 123 nm were found to cause the release of more pro-inflammatory cytokines compared to smaller particles [199]. Cationic liposomes showed a better adjuvant effect compared to neutral and anionic liposomes. They can activate the complement system and are easily cleared by macrophages [200]. Aggregated liposomes were also found to trigger the complement system [201]. 

Our group synthesized phospholipid-coated AgNPs which were then immobilized onto plasma-coated solid surfaces. In addition to excellent antibacterial properties, we found a reduction in the level of expression of pro-inflammatory cytokines from bone marrow-derived macrophages, thereby alleviating the inflammatory response [77]. Several other lipids conjugated of silver nanomaterials were also reported, such as those with dipalmitoyl phosphatidylcholine (DPPC) [202], phosphatidylglycerol [203], cholesterol [204], egg lecithin [203], phospholipids [205], etc. However, the impact on the immune system of those lipid-protected NPs has yet to be explored. Despite the promise of AgNPs conjugated with biological molecules, very little has been done to fully explore how this approach can be used to modulate the inflammatory response. This is an area that warrants much research efforts in the years to come. 

## 6. Conclusions

In this review article, we provide a critical overview of the current state of knowledge of the effect of silver nanomaterials on inflammatory responses. The potent antibacterial properties of silver, silver-based coatings, and silver nanomaterials have inspired a tremendous amount of research and commercial products that are now available on the market. Some of these products are used in hospitals and protect patients from deadly infections. One aspect that has been insufficiently explored is the effect of silver on the immune system. The available literature reveals that immunological consequences depend on many factors, including the size, shape, surface charge, and surface chemistry of the nanomaterial. These characteristics are all capable of modulating the way inflammatory cells such as neutrophils and macrophages recognize and perceive a nanoscale object. However, a clear correlation between these characteristics is impossible to draw at present. This review emphasizes the pro-inflammatory, anti-inflammatory, and adjuvant properties of silver nanomaterials, highlighting the mechanisms by which the immune system interacts with these foreign materials.

We are now at the crossroad when further advances in the use of silver in medicine require an understanding of the immunological events associated with the application of silver (nano)materials. This will allow for the rational design of the next generation of intelligent approaches which benefit not only from the known capacity of silver to kill pathogenic bacteria but also the potential to extract desired inflammatory responses that can stimulate events such as faster wound healing and better tissue regeneration. Bio-inspired shielding of silver nanomaterials is an interesting approach to design nanomaterials with immunomodulatory properties. Ornamenting the silver nanomaterials with biomolecules can prevent undesirable binding of proteins which normally signal the immune system about a foreign entry. Despite the promises of this versatile approach, collaborative attempts from across disciplines are necessary to meet the existing knowledge gaps and to fully exploit the potential of silver nanomaterials in future clinical applications. 

## Figures and Tables

**Table 1:** In vitro evaluation of the immune response of silver nanomaterials.

Type of Ag	Size	Reagents Used	Type of Immune Cells	Cytokines Expression	In vitro Inflammatory Assays	Ref
**NPs**	<20 nm	AgNO_3_, Quercetin, Polyoxyethylene Glycerol trioleate, and Tween 20	Caco-2 cells	Decreased IL-8 expression	qRT-PCR, ELISA, total protein content, Nitrate/Nitrite Colorimetric Assay	[68]
**Nano wires**	10 µm	AgNO_3_, ethylene glycol, poly (vinylpyrrolidone)	Human monocyte-derived macrophages	Up taken by macrophages and transformed to silver chloride	High angle annular dark field scanning electron microscopy, Confocal analysis	[69]
**Nanoclusters**	1.5 nm	NaBH_4_, AgNO_3_	RAW264.7 cells	Release TNF-α, IL-6	ELISA	[70]
**NPs**	14 nm	NaBH_4_, AgNO_3_, Sodium citrate	RAW264.7 and J774.1	Reduced TNF-α expression	ELISA	[71]
**NPs**	10–50 nm	AgNO_3_, Extracts of *Viburnum opulus*	Hacat cells	Increased IL-1α and decreased IL-1α, IL-6	ELISA	[72]
**NPs**	20–80 nm	AgNO_3_, Extracts of *Sambucus nigra*	Hacat cells	Reduced IL-1α production	ELISA	[73]
**NPs**	10 nm	Dendrimer, NaBH_4_, AgNO_3_, Sodium citrate	RAW264.7 and J774.1	Decreased TNF-α, IL-6	ELISA	[74]
**NPs**	23.52–60.83 nm	AgNO_3_, Ethanolic petal extract of *Rosa indica*	Rat peritoneal macrophages	Attenuate production of NO and superoxide	Nitrate/Nitrite Colorimetric Assay, Estimate superoxide anion generation	[75]
**NPs**	10.29–45.57 nm	AgNO_3_, Aqueous extracts of *Phyllanthus acidus* L.	Rat peritoneal macrophages	Attenuate production of IL-1α, NO and superoxide	ELISA, Immunoblotting, Nitrate/Nitrite Colorimetric Assay, Estimate superoxide anion generation	[76]
**NPs**	4 nm	Chloroform, NaBH_4_, AgNO_3_, POPS	Bone marrow-derived macrophage cells	Decrease in IL-6 and IL-1β, no effect in TNF-α	ELISA	[77]

**Table 2:** In vivo assessment of the immune response of silver nanomaterials.

Nature of Ag	Size	Reducing Agent Used	Animal Strain	Model	Outcome	Ref
**NPs**	9.3 ± 3.2 nm	NaBH_4_, AgNO_3_, Sodium citrate	Balb/c mice	Postoperative adhesion model	Decrease inflammation in peritoneal adhesion without toxic effects	[71]
**Nano** **wires**	1.5 µm and 10 µm	AgNO_3_, ethylene glycol, polyvinyl pyrrolidone	Sprague Dawley rats	Intratracheal instillation, Lung model	Completely internalized by lung macrophages with toxic effects	[78]
**NPs**	7–10 nm	AgNO_3_, Leaf extracts of Terminalia species	Wistar albino rats	Hind paw oedema model	Inhibition of oedema by 95%	[79]
**NPs**	10–50 nm	AgNO_3_, Extracts of *Viburnum opulus* L.	Wistar rats	Carrageenan-induced inflammation models	Decreased inflammation	[72]
**NPs**	14 ± 9.8 nm	NaBH_4_, AgNO_3_, Sodium citrate	Male Balb/c mice	Thermal injury animal models	Silver can modulate cytokine expression	[80]
**NPs**	10 nm (5–15 nm)	Dendrimer, NaBH_4_, AgNO_3_, Sodium citrate	C57BL/6 N mice	Excisional and burn wound models	Enhanced anti-inflammatory efficacy	[74]
**NPs**	20–80 nm	AgNO_3_, Extracts of *Sambucus nigra*	Male Wistar rats,	Carrageenan-induced inflammation models	AgNPs enhanced inflammation edema rate	[73]
**NPs**	12–22 nm	Starch, NaOH, AgNO_3_, Absolute ethanol	Male and female rats	Grade II burn wound models	Reduce rat paw oedema	[81]
**Nano crystalline silver**	10–15 nm	AgNO_3_, polyethene	Domestic White/Landrace swine	Porcine contact dermatitis model	Treated normal pigs have near-normal skin after 24 h	[82]
**Silver-coated glass beads**	850–1400 µm and 5 µm	Borosilicate glass beads	Male Balb/c mice	Models mimicking Crohn’s disease and ulcerative colitis	Attenuated inflammation in colitis and Crohn’s disease models	[83]
**NPs**	7 ± 3 nm	AgNO_3_, Diaminopyridiinyl Heparin, Glucose,	Male rats	Carrageenan-induced paw edema	Localization of anti-inflammatory effects	[84]
